# Hippocampal Regulation of Postsynaptic Density Homer1 by Associative Learning

**DOI:** 10.1155/2017/5959182

**Published:** 2017-11-07

**Authors:** Nicholas E. Clifton, Darren Cameron, Simon Trent, Lucy H. Sykes, Kerrie L. Thomas, Jeremy Hall

**Affiliations:** ^1^Neuroscience and Mental Health Research Institute, Cardiff University, Cardiff CF24 4HQ, UK; ^2^MRC Centre for Neuropsychiatric Genetics and Genomics, Institute of Psychological Medicine and Clinical Neurosciences, Cardiff University, Cardiff CF24 4HQ, UK; ^3^School of Biosciences, Cardiff University, Cardiff CF24 4HQ, UK

## Abstract

Genes involved in synaptic plasticity, particularly genes encoding postsynaptic density proteins, have been recurrently linked to psychiatric disorders including schizophrenia and autism. Postsynaptic density Homer1 proteins contribute to synaptic plasticity through the competing actions of short and long isoforms. The activity-induced expression of short *Homer1* isoforms, *Homer1a* and *Ania-3*, is thought to be related to processes of learning and memory. However, the precise regulation of *Homer1a* and *Ania-3* with different components of learning has not been investigated. Here, we used in situ hybridization to quantify short and long *Homer1* expression in the hippocampus following consolidation, retrieval, and extinction of associative fear memory, using contextual fear conditioning in rats. *Homer1a* and *Ania-3*, but not long *Homer1*, were regulated by contextual fear learning or novelty detection, although their precise patterns of expression in hippocampal subregions were dependent on the isoform. We also show for the first time that the two short Homer1 isoforms are regulated after the retrieval and extinction of contextual fear memory, albeit with distinct temporal and spatial profiles. These findings support a role of activity-induced Homer1 isoforms in learning and memory processes in discrete hippocampal subregions and suggest that Homer1a and Ania-3 may play separable roles in synaptic plasticity.

## 1. Introduction

The *Homer1* gene codes for a family of postsynaptic density proteins with key roles in the control of synaptic plasticity [1–4] and learning and memory [5–7]. In humans and other mammals, several *Homer1* isoforms exist, most of which are long and constitutively expressed whilst two shorter isoforms, *Homer1a* and *Ania-3*, act as activity-induced immediate early genes [8, 9]. Both long and short Homer1 proteins share the same N-terminal binding domain and have common targets [10]. However, upon the recruitment of short Homers to the postsynaptic density, they disrupt the interactions between long Homers and their target proteins through dominant negative regulation [11, 12]. Previous studies have shown that, by inhibiting the actions of long Homer isoforms, Homer1a and Ania-3 regulate metabotropic glutamate receptor (mGluR) function [13–15] and calcium homeostasis [16–19].

Alterations of short Homer1 expression affect synaptic strength. Overexpression of Homer1a in the hippocampus induces a glutamate-independent modulation of surface AMPA receptors [2, 20–22], reduced GluA2 subunit tyrosine phosphorylation [2], and blockade of long-term potentiation [21, 23]. The ratio of short to long Homer1 in dendritic spines is a key mediator of AMPA currents [24, 25]. Further studies have also shown that hippocampal Homer1a overexpression impairs spatial working and reference memory [6, 23], whilst knocking out Homer1 short forms cause deficits in fear conditioning [7, 26]. However, the respective roles of Homer1a and Ania-3 in learning and memory have not been distinguished.

In genetic studies of schizophrenia and other psychiatric disorders including autism, converging evidence points to postsynaptic density proteins in their aetiology [27–31], particularly those involved in the regulation of associative learning [32, 33]. Postmortem analyses show that Homer1 protein levels are altered in patients with psychiatric disorders [34, 35]. A recent study observed a decrease in long Homer1 isoforms but increased short-form Homer1a in postmortem hippocampal CA1 from patients with schizophrenia [36], suggesting that these patients had a higher ratio of short to long Homer1 proteins in this region. Furthermore, *Homer1a* and *Ania-3* are induced rapidly by psychoactive compounds [37], such as ketamine [38, 39] and cocaine [1, 40, 41].

Homer1 activity has been shown to influence the functioning of other proteins and protein complexes linked to psychiatric disorders through genetic variants, such as FMRP [28, 42, 43], CYFIP1 [27, 44, 45], Arc [27, 46], SHANK [10, 47, 48], and the calcium channel Ca_v_1.2 [16, 42]. Genetic variants within the *HOMER1* gene itself have been associated with schizophrenia in some studies [49, 50], and whilst more recent genome-wide association studies have not found a significant link between *HOMER1* SNPs and schizophrenia, many of the interactors of Homer1 have been robustly associated with the disorder [42]. Furthermore, mutations in HOMER1 have also been associated with other psychiatric disorders including autism [51–53].

Whilst there has been substantial investigation of the induction of Homer1 isoforms following exposure to psychoactive compounds [38–41, 54] and environmental stressors [55, 56] in rodents, fewer studies have focused on its expression after learning. Hernandez et al. demonstrated that the corticostriatal expression pattern of *Homer1a* varies with successive trial of instrumental learning [57]. More recently, Mahan et al. reported that de novo *Homer1a* expression occurs in the hippocampus and amygdala of mice following fear conditioning [7]. However, examination of *Homer1* expression following constitutive components of associative learning has not yet been reported and no studies have examined the differential activation of the short Homer isoforms *Homer1a* and *Ania-3* during learning.

In order to explore the contribution from activity-induced Homer1 proteins to different components of associative fear learning, we quantified the respective expression patterns of *Homer1* isoforms after the consolidation, retrieval, and extinction of conditioned fear memories using a single-trial contextual fear conditioning paradigm [58, 59]. In addition, to enable further interrogation of the precise role of these proteins in memory consolidation, we employed a protocol which separates the contextual fear conditioning paradigm into its constitutive parts. In the context pre-exposure facilitation effect (CPFE) protocol, the processes of learning about the context and associating the contextual memory with a footshock occur during separate, consecutive training events. Since context encoding is necessary for context conditioning [60, 61], only rats preexposed to the context that they are subsequently given an immediate shock in undergoing associative fear learning pertaining to that context. Thus, immediate early gene expression changes resulting from exposure to nonspecific aspects of fear conditioning versus the context-shock association can be quantified independently [60, 62].

## 2. Materials and Methods

### 2.1. Subjects

The subjects were 120 adult male Lister hooded rats (Charles River, UK) weighing 275–325 g. Rats were housed in pairs with food and water access ad libitum. The holding room was maintained at 21°C with a 12 h reversed light/dark cycle. Subjects were given at least 5 days to acclimatise to the holding room prior to testing. The handling of animals from each experimental group was ordered pseudorandomly. All procedures were conducted in accordance with the local Cardiff University Ethical Committee approval and the United Kingdom 1986 Animals (Scientific Procedures) Act.

### 2.2. Contextual Fear Conditioning

Rats underwent a contextual fear conditioning paradigm [63, 64]. In conditioning training trials, rats were individually exposed to a novel context (conditioning chamber; conditioned stimulus (CS)) for 3 min. At 2 min, rats received an unconditioned stimulus (US) consisting of a single scrambled footshock (0.5 mA, 2 sec). Freezing behaviour was quantified pre- and post-US as an index of conditioned fear. Rats were returned to their home cages immediately after conditioning and were killed by CO_2_ inhalation 30 min, 2 h, 4 h, or 24 h after conditioning. Control subjects were naïve littermates killed at the same time of day as conditioned animals.

To compare the effects of the fear conditioning paradigm with solely the exposure to a novel context, rats were placed in a novel context for 3 min without the administration of a footshock and killed by CO_2_ inhalation 30 min afterwards. These were compared to rats which had undergone a conditioning training trial and killed at the same time point. Freezing behaviour was quantified throughout the duration of context exposure.

Subjects from retrieval or extinction experimental groups underwent conditioning followed by a short or long recall trial, respectively. Recall trials took place 48 h after conditioning. Rats were reexposed to the conditioned context for 2 min (short recall; insufficient to produce extinction [64]) or 10 min (long recall, sufficient to produce extinction [64]) before being returned to home cages. Freezing behaviour during recall trials was quantified. Rats were killed 30 min or 2 h after recall trials. Control “no recall” subjects underwent a conditioning training trial and were killed 48 h afterwards.

Whole brains were immediately dissected and snap frozen on dry ice before storage at −80°C until use in in situ hybridization.

### 2.3. Context Preexposure Facilitation Effect

In an immediate shock paradigm, rats were exposed to one of two different contexts, context A or context B, for 20 min/day for three consecutive days, to familiarise the animals to one context. On the fourth day, rats were placed into either the familiar context or the novel context. Subjects of the context pre-exposure facilitation effect (CPFE) group were placed into the familiar context and received an immediate footshock (0.5 mA, 2 sec) before immediate removal. These were compared to two groups placed into the novel context for the same short duration: one group received an immediate footshock (Novelty IS) and the other group did not (Brief Novelty). Rats were killed by CO_2_ inhalation 30 min after the final context exposure. A further group of naïve littermates was killed by CO_2_ inhalation at the same time of day. Whole brains were rapidly dissected and snap frozen on dry ice before storage at −80°C.

### 2.4. In Situ Hybridization

In situ hybridization is a method of localising and quantifying specific mRNA sequences in fixed tissue sections by hybridising with labelled strands of complementary nucleotide sequences. Coronal brain sections (14 *μ*m) containing dorsal regions of the hippocampus were cut, thaw mounted onto poly-L lysine-coated glass slides, and fixed in 4% paraformaldehyde prior to dehydration in ethanol and storage in 95% ethanol at 4°C. Oligonucleotide probes were designed to target specific transcripts of the *Homer1* gene: *Homer1a*, 5′-CATGATTGCTGAATTGAATGTGTACCTATGTGAAAATGGCAATGC-3′; *Ania-3*, 5′-GGTAGGGCGGAGGATTCATGACAGACAATACATGAACTTGGGCAG-3′. Long forms of *Homer1* were targeted as a group with one oligonucleotide probe: *Homer1b/c/f/g*, 5′-CTCTGTCTTGTGGCTGTGCACCGCGTTTGCTTGACTACTAACACA-3′. *Arc* expression was also assessed for comparison: *Arc,* 5′-AGCATCTCAGCTCGGCACTTACCAATCTGCAGGATCACATTGGGT-3′. Oligonucleotide probes (Sigma-Aldrich) were 3′-end-labelled with [*α*-^35^S] dATP (PerkinElmer) then hybridized [65] to tissue sections matched for the hippocampal region across subjects. For each subject, two technical replicates were used. As a negative control, a third section was incubated with 100x excess of unlabelled probe. Autoradiographs were generated using radiographic film exposed to the sections for 5–10 days (probe dependent) and developed. Autoradiograph densitometry in three hippocampal subregions (*Cornu Ammonis* 1 (CA1), CA3, and dentate gyrus (DG)) was quantified as a measure of mRNA concentration.

### 2.5. Statistical Analysis

Percent freezing was compared within subjects (learning phase) and between groups (euthanasia time) using two-way repeated measures analysis of variance (ANOVA). Main effects of behavioural intervention on mRNA expression within each hippocampal subregion were determined using independent one-way ANOVA tests. Post hoc Dunnett's multiple comparison procedure was applied to data which surpassed a significance threshold (alpha = 0.05) in ANOVA, to determine specific group differences and directionality.

## 3. Results

### 3.1. Determining the Regulation of *Homer1* Expression by Contextual Fear Memory Conditioning

**Figure 1:**
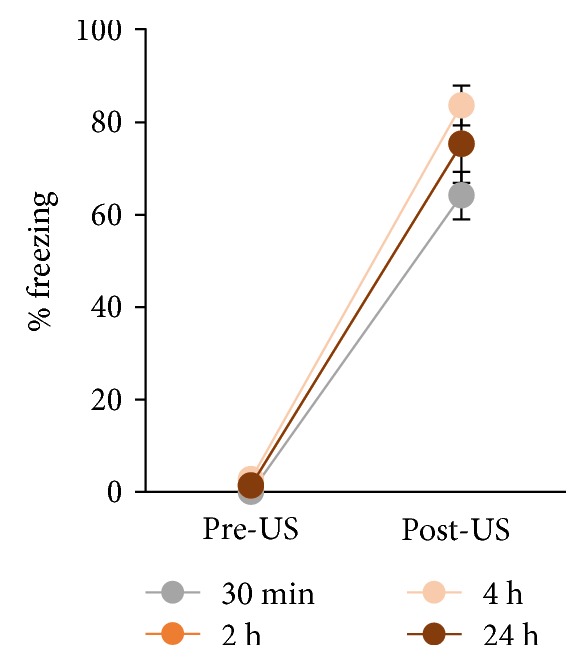
Contextual fear conditioning induced robust postshock freezing. Rats were exposed to a novel context for 3 min and received a footshock after 2 min. Data representative of all experimental groups culled 30 min, 2 h, 4 h, and 24 h after conditioning for in situ hybridization (*n* = 6 per group). Data represented by mean ± SEM percent freezing.

To investigate memory consolidation-induced gene expression of *Homer1*, rats underwent a conditioning training trial. During contextual fear conditioning training, rats displayed robust postshock freezing, compared to baseline (*P* < 0.001, two-way repeated measures ANOVA; Figure 1). Brains were extracted from separate groups 30 min, 2 h, 4 h, and 24 h after conditioning for mRNA quantification, to observe the regulation of mRNA species over time.

**Figure 2:**
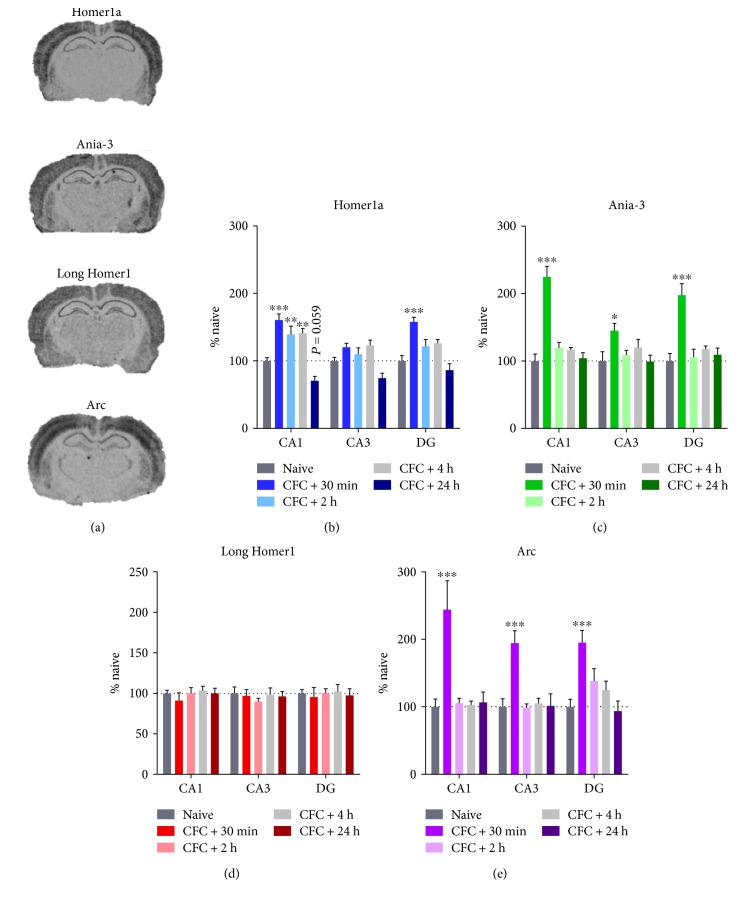
(a) Representative autoradiographs of coronal rat brain sections (−3.5 mm posterior to the bregma) hybridized with oligonucleotide probes targeting Homer1a, Ania-3, long Homer1, or Arc. (b–e) Hippocampal mRNA expression of (b) Homer1a, (c) Ania-3, (d) long Homer1, and (e) Arc following contextual fear conditioning (CFC). mRNA expression from the CA1, CA3, and dentate gyrus (DG) regions is represented by mean ± SEM standardised optical density values, normalised to naïve controls (100%). Brains were dissected 30 min, 2 h, 4 h, or 24 h after conditioning. *n* = 6 per group. Asterisks represent significance of the groups versus naïve control (^∗^*P* < 0.05, ^∗∗^*P* < 0.01, and ^∗∗∗^*P* < 0.001).

Each *Homer1* isoform was strongly expressed throughout all hippocampal areas observed, with the exception of weak *Homer1a* expression in the dentate gyrus (Figure 2(a)). These expression patterns are consistent with those observed previously using similar methods [66].

Analysis of the expression of short *Homer1* isoforms following contextual fear conditioning revealed a main effect on *Homer1a* and *Ania-3* in each hippocampal subregion (*Homer1a*: CA1 F_(4,25)_ = 19.47, *P* < 0.001; CA3 F_(4,25)_ = 7.13, *P* < 0.001; and DG F_(4,25)_ = 11.25, *P* < 0.001 and *Ania-3*: CA1 F_(4,25)_ = 27.08, *P* < 0.001; CA3 F_(4,25)_ = 3.03, *P* < 0.05; and DG F_(4,25)_ = 12.23, *P* < 0.001; one-way ANOVA, Figures 2(b) and 2(c)). The expression of long *Homer1* isoforms, as indicated by a pan-*Homer1b/c/f/g* oligonucleotide probe, was unchanged in all regions (CA1 F_(4,25)_ = 0.47, *P* = 0.75; CA3 F_(4,25)_ = 0.31, *P* = 0.87; and DG F_(4,25)_ = 0.10, *P* = 0.98; one-way ANOVA, Figure 2(d)). *Arc* expression, which has previously been shown to be coordinated with *Homer1a* in hippocampal circuits [46, 67], was also affected by contextual fear conditioning in each subregion (CA1 F_(4,25)_ = 8.69, *P* < 0.001; CA3 F_(4,25)_ = 9.81, *P* < 0.001; and DG F_(4,25)_ = 6.93, *P* < 0.001; one-way ANOVA, Figure 2(e)). Post hoc Dunnett's tests indicated that *Homer1a* expression was increased in the CA1 region at 30 min, 2 h, and 4 h after conditioning and in the dentate gyrus at 30 min only. No changes in CA3 *Homer1a* expression were revealed in post hoc tests. In contrast, *Ania-3* and *Arc* expression was increased in all three hippocampal regions, yet solely at the 30 min time point (Figure 2).

### 3.2. The Regulation of Short *Homer1* Isoforms by Novel Context Exposure

**Figure 3:**
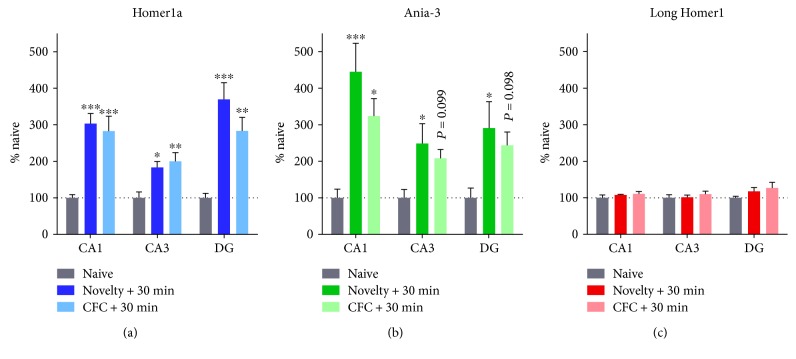
mRNA expression of (a) Homer1a, (b) Ania-3, and (c) long Homer1 following exposure to a novel context or contextual fear conditioning (CFC). mRNA expression is represented by mean ± SEM standardised optical density values, normalised to naïve controls (100%). Brains were dissected 30 min after context exposure. *n* = 6 per group. Asterisks represent significance of the groups versus naïve control (^∗^*P* < 0.05, ^∗∗^*P* < 0.01, and ^∗∗∗^*P* < 0.001).

The previous experiments show that both *Homer1a* and *Ania-3* are induced in the hippocampus by contextual fear conditioning. Since this procedure involves the exposure of the animal to a novel context and past studies have shown *Homer1a* to be upregulated by novelty alone [46], we also investigated the expression of *Homer1a* and *Ania-3* 30 min after novel context exposure compared to fear conditioning in separate cohorts. The expression of both short *Homer1* isoforms was modified by context exposure at this time point (*Homer1a*: CA1 F_(2,15)_ = 15.42, *P* < 0.001; CA3 F_(2,15)_ = 7.75, *P* < 0.01; and DG F_(2,15)_ = 15.57, *P* < 0.001 and *Ania-3*: CA1 F_(2,15)_ = 10.25, *P* < 0.01; CA3 F_(2,15)_ = 4.33, *P* < 0.05; and DG F_(2,15)_ = 4.13, *P* < 0.05; one-way ANOVA) but not the long *Homer1* isoforms (CA1 F_(2,15)_ = 0.83, *P* = 0.46; CA3 F_(2,15)_ = 0.49, *P* = 0.62; and DG F_(2,15)_ = 1.55, *P* = 0.24; one-way ANOVA, Figure 3). Post hoc Dunnett's tests were used to determine whether novel context exposure alone is sufficient to induce expression of the short *Homer1* isoforms. Similarly to that seen previously, *Homer1a* and *Ania-*3 appeared to be induced at 30 min by contextual fear conditioning in each hippocampal subregion, although there was only a trend (*P* < 0.1) to an increase in *Ania-*3 expression in CA3 and the dentate gyrus in this experiment. Novel context exposure significantly increased *Homer1a and Ania-3* expression in all regions. In all regions, there was no difference between the effects of conditioning or novel context exposure on the expression of *Homer1a*. There was no change in the expression of *Long Homer1* with novelty or CFC training in any hippocampal subfield (Figure 3).

### 3.3. The Regulation of *Homer1* Expression Using the Context Preexposure Facilitation Effect (CPFE) Behavioural Model of Contextual Fear Conditioning

**Figure 4:**
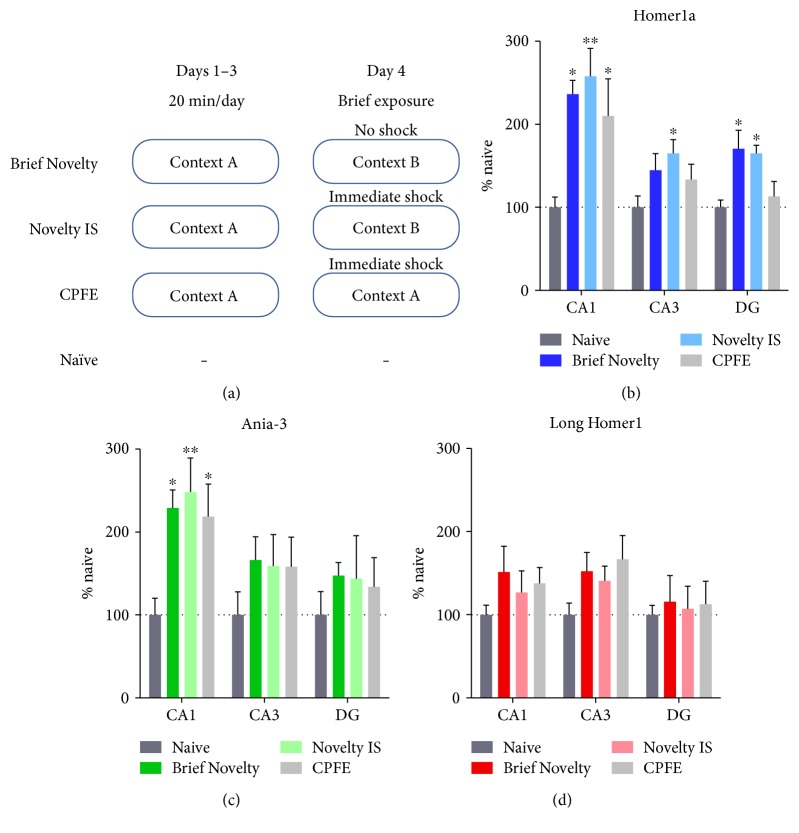
(a) Schematic displaying the context preexposure facilitation effect (CPFE) behavioural protocols. (b–d) Hippocampal mRNA expression of (b) Homer1a, (c) Ania-3, and (d) long Homer1 following a brief exposure to a novel context (Brief Novelty), brief novel context exposure with an immediate footshock (Novelty IS), and brief exposure to a familiar context with an immediate footshock (CPFE). mRNA expression is represented by mean ± SEM standardised optical density values, normalised to naïve controls (100%). Brains were dissected 30 min after the final context exposure. *n* = 6 per group. Asterisks represent significance of the groups versus naïve control (^∗^*P* < 0.05, ^∗∗^*P* < 0.01).

To further examine whether *Homer1a* or *Ania-3* are specifically induced by associative events (CS-US and context memory formation) or by the processes associated with the standard contextual fear conditioning paradigm such as novel stimulus presentation, we used a context preexposure facilitation effect (CPFE) procedure to further dissect the regulation of short *Homer1* isoforms by associative fear learning. Training was split into three components: brief exposure (14–20 s) to a novel context with no presentation of the US (Brief Novelty); immediate footshock given upon exposure to a novel context (Novelty IS); and brief exposure to a familiar context with an immediate footshock (CPFE). Only the CPFE procedure is sufficient to generate an associative context-footshock fear memory [60–62]. Since we anticipated the time course of any induction in short *Homer1* gene expression to be similar to that observed following contextual fear conditioning previously, brains were only extracted 30 min after each behavioural intervention. There was a main effect of the group on *Homer1a* and *Ania-3* expression within the CA1 region of the hippocampus (*Homer1a*: F_(3,20)_ = 5.53, *P* < 0.01; and *Ania-3*: F_(3,20)_ = 4.60, *P* < 0.05; one-way ANOVA, Figures 4(b) and 4(c)). *Homer1a* expression was also modified in the dentate gyrus (F_(3,20)_ = 5.19, *P* < 0.01), but was not significantly modified in CA3 (F_(3,20)_ = 2.47, *P* = 0.092). *Ania-3* expression was unchanged in CA3 (F_(3,20)_ = 0.89, *P* = 0.46) and DG (F_(3,20)_ = 0.38, *P* = 0.77). Post hoc Dunnett's tests on data from CA1 revealed that, compared to naïve controls, the expression of both *Homer1a* and *Ania-3* was increased in the CA1 region following each stimulus, irrespective of context-footshock pairing, implying that the induction of short *Homer1* isoforms did not depend on associative learning per se. There was a significant increase in the expression of *Homer1a* in CA3 and the dentate gyrus in the Novelty IS group but not the CPFE group. Thus, there is a lack of correlation in the regulation of *Homer1a* with associative CS*-*US learning in these two hippocampal subregions. In contrast, there were no significant changes in the expression of *Ania-3* in either the CA3 or dentate gyrus with behavioural training. Therefore, there is dissociation between the transcriptional regulation of *Homer1a* and *Ania-3* expression with associative learning in the hippocampal CA3 and dentate gyrus.

Consistent with expression in the standard contextual fear conditioning protocol, the levels of *Long Homer1* mRNA remained unchanged in all groups (CA1 F_(3,20)_ = 0.88, *P* = 0.47; CA3 F_(3,20)_ = 1.79, *P* = 0.18; and DG F_(3,20)_ = 0.072, *P* = 0.97; one-way ANOVA, Figure 4(d)).

### 3.4. Expression of Short and Long *Homer1* Isoforms following Contextual Fear Memory Retrieval and Extinction

**Figure 5:**
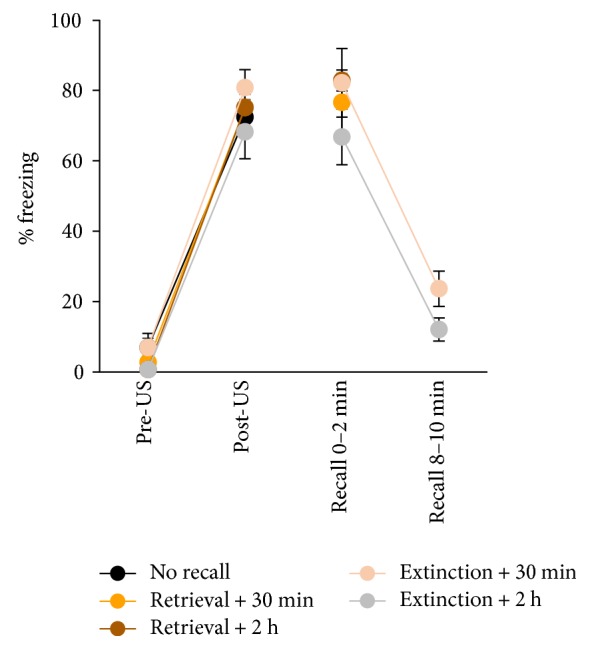
In the retrieval and extinction of contextual fear memory, rats displayed robust conditioned freezing and an extinguished response after long recall (10 min). Retrieval and extinction training took place 48 h after conditioning. Control “no recall” animals were conditioned and culled 48 h afterwards. Rats were culled 30 min or 2 h after retrieval and extinction for in situ hybridization. *n* = 6 (30 min), *n* = 12 (2 h), or *n* = 18 (no recall) per group. Data represented by mean ± SEM percent freezing.

**Figure 6:**
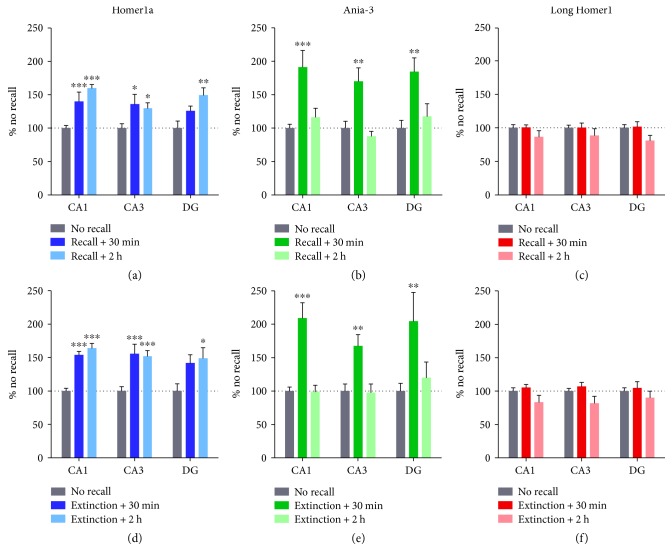
mRNA expression of Homer1a, Ania-3, and long Homer1 in the hippocampus following the recall (a–c) or extinction (d–f) of contextual fear memory. mRNA expression is represented by mean ± SEM standardised optical density values, normalised to no recall controls (100%). Brains were taken 30 min or 2 h after reexposure. *n* = 6 (30 min), *n* = 12 (2 h), or *n* = 18 (no recall) per group. Asterisks represent significance of the groups versus no recall control (^∗^*P* < 0.05, ^∗∗^*P* < 0.01, and ^∗∗∗^*P* < 0.001).

In order to investigate whether short *Homer1* isoforms are induced in the hippocampus by other associative learning processes, gene expression was quantified after the retrieval and extinction of contextual fear. All groups trained using the standard fear conditioning protocol displayed robust postshock freezing (F_(1,31)_ = 492.9, *P* < 0.001, two-way repeated measures ANOVA) and extinction training induced a within-trial reduction of freezing (F_(1,10)_ = 115.1, *P* < 0.001, two-way repeated measures ANOVA; Figure 5). Reexposure to the context for 2 min (retrieval), which is not sufficient to induce extinction [64], modified the expression of *Homer1a* (CA1 F_(2,33)_ = 28.78, *P* < 0.001; CA3 F_(2,33)_ = 4.98, *P* < 0.05; and DG F_(2,33)_ = 5.61, *P* < 0.01; one-way ANOVA) and *Ania-3* (CA1 F_(2,33)_ = 11.64, *P* < 0.001; CA3 F_(2,33)_ = 9.04, *P* < 0.001; and DG F_(2,33)_ = 5.08, *P* < 0.05; one-way ANOVA) compared to “no recall” control subjects (Figure 6). A 10 min recall trial (extinction) also affected the expression of both short *Homer1* isoforms across hippocampal subregions (*Homer1a*: CA1 F_(2,33)_ = 38.83, *P* < 0.001; CA3 F_(2,33)_ = 13.82, *P* < 0.001; and DG F_(2,33)_ = 4.47, *P* < 0.05 and *Ania-3*: CA1 F_(2,33)_ = 24.00, *P* < 0.001; CA3 F_(2,33)_ = 5.91, *P* < 0.01; and DG F_(2,33)_ = 4.70, *P* < 0.05; one-way ANOVA). A significant retrieval-induced upregulation of *Homer1a* occurred at 30 min and 2 h in CA1 and CA3 and at 2 h only in the dentate gyrus. *Ania-3* expression was upregulated at 30 min, but not 2 h, in all three hippocampal regions. The hippocampal expression pattern of each short *Homer1* isoform following extinction learning mirrored that following retrieval. Long *Homer1* expression was unchanged by context reexposure in any of the hippocampal subregions observed (retrieval: CA1 F_(2,21)_ = 1.40, *P* = 0.27; CA3 F_(2,21)_ = 0.91, *P* = 0.42; and DG F_(2,21)_ = 2.60, *P* = 0.098 and extinction: CA1 F_(2,21)_ = 2.42, *P* = 0.11; CA3 F_(2,21)_ = 3.22, *P* = 0.060; and DG F_(2,21)_ = 0.79, *P* = 0.47; one-way ANOVA).

## 4. Discussion

The present findings indicate that the expression of short *Homer1* isoforms in the hippocampus is correlated with experiencing a salient context, which may contribute to the formation or maintenance of Pavlovian associations. Within this, the relative expression of *Homer1a* and *Ania-3* in different subregions of the hippocampus depended on the type of memory processing involved. Crucially, in some instances, the region-specific regulation of *Homer1a* differed to that of *Ania-3*. In addition, the temporal profiles of *Homer1a* and induction differed, with *Ania*-*3* expression having a more curtailed profile. Long forms of *Homer1*, which were targeted as a group with one oligonucleotide probe, were not regulated by contextual fear conditioning, retrieval, or extinction or stimulus-driven experience in any hippocampal subregion or time point measured, consistent with its previously reported constitutive expression [8].

Homer1a functions to potentiate synaptic transmission [22] and is required for the formation of fear memory [26]. The induction of short *Homer1* isoforms in the CA1 is consistent with its role as a key region for the consolidation of contextual fear (CS-US) memory [68, 69]. Moreover, the hippocampus plays an important role in novelty detection [70–73]. Our data show that the expression of *Homer1a* and *Ania*-*3* in CA1 is also driven by novelty. The direct monosynaptic pathway from the entorhinal cortex conveys inputs from sensory systems to CA1 and to serve novelty detection [74]. As such, the regulation of short *Homer1* isoforms may correlate with the contribution of novelty to the comparator function of CA1 and the encoding of memory [75].

The regulation of *Homer1a* and *Ania-3* in the CA3 and dentate gyrus after contextual fear conditioning and novelty exposure is more likely to be related to the Pavlovian associative events of contextual memory formation. The formation of conjunctive spatial or contextual representations is necessary for the formation of an association between a stimulus and a context (CS-US association) [76–81]. However, the absence of transcriptional activation in the CPFE group negates regulation in these regions correlated with CS-US formation. The increases in both *Homer 1a* and *Ania*-*3* expression in the dorsal CA3 and dentate gyrus are thus consistent with the role of these hippocampal subregions in the formation of contextual representation [82, 83]. The pattern of transcriptional induction in the Novelty IS and Brief Novelty experimental groups, characterised by very short stimulus presentations, suggests that specific processes underlying arousal or attentional processes accompanying aversive footshock US and novel CS detection additionally regulate the expression of *Homer1a*, but not *Ania-3*, in CA3 and the dentate gyrus. However, the observation of short *Homer1* expression at additional time points may reveal a different pattern of induction. The possibility that *Ania-3* may have a more restrictive role in the formation of contextual memory also demands further investigation.

The regulation of *Homer1a* and *Ania-3* expression after contextual fear conditioning exhibited striking temporal differences. This was reflected in the duration for which the elevated expression level was detectable. Regulated *Ania-3* expression was considerably more transient than *Homer1a* in all hippocampal subregions. This observation, which is previously unreported, implies that these two activity-induced isoforms are regulated through different transcriptional and/or posttranscriptional mechanisms. It is possible that this discrepancy occurs due to differences in metabolic processing, yet may also have implications for the function of the translated proteins. Coincident regulation of *Homer1a* and *Arc* in the hippocampus has been previously reported [46]. Whilst we show similar coincidental regional induction of *Homer1a* and *Arc* with contextual fear conditioning, in terms of a temporal profile, the expression of *Ania-3* more closely parallels the expression of Arc. Based on the current observations, it may be hypothesised that whilst these synaptic proteins may function in concert in the structural and synaptic modifications supporting memory formation, there is a closer association of Ania-3 and Arc*-*mediated processes.

We observed an increase in the expression of both *Homer1a* and *Ania-3* in all three hippocampal subfields after conditioned context cued recall, whether the recall period was short (2 min) or long (10 min). These changes reflect the coordinated activity of the hippocampal subregions in the retrieval of contextual fear memory [84, 85]. Similar increases have been observed for other immediate early genes including Arc [86–88]. The transcriptional activation of the short *Homer1* isoforms after contextual fear memory retrieval may be related to the hippocampal-dependent memory processes of reconsolidation and extinction associated with short and prolonged conditioned context exposure, respectively [63, 89, 90]. These findings encourage the study of the contribution of Homer1a and Ania-3 to post retrieval plasticity mechanisms, particularly with regard to their roles in distinct hippocampal subregions that may themselves differentially contribute to extinction and reconsolidation [84, 85, 91–93]. This is the first time that evidence for a role of short *Homer* isoforms in retrieval-related memory processes, including extinction learning, has been presented. It also implicates Homer1 in specific cognitive processes relevant to psychiatric disorders [33].

## 5. Conclusions

In conclusion, the present results build on previous studies showing that short, activity-induced Homer1 immediate early genes are involved in learning and memory. We present novel findings showing that these short *Homer1* variants are expressed not only following the consolidation of contextual fear memories but also accompanying their retrieval and extinction. We also demonstrate for the first time that *Homer1a* and *Ania-3* are differentially regulated both temporally and spatially in the hippocampus during fear learning processes and therefore may have different roles in the regulation of long-term contextual fear-memory associations.
